# Iterative Bounded Synthesis for Efficient Cycle Detection in Parametric Timed Automata

**DOI:** 10.1007/978-3-030-72016-2_17

**Published:** 2021-03-01

**Authors:** Étienne André, Jaime Arias, Laure Petrucci, Jaco van de Pol

**Affiliations:** 8 Eindhoven University of Technology, Eindhoven, The Netherlands; 9 Aalborg University, Aalborg East, Denmark; 10 Université de Lorraine, CNRS, Inria, LORIA, Nancy, France; 11 LIPN, CNRS UMR 7030, Université Sorbonne Paris Nord, Villetaneuse, France; 12 Aarhus University, Aarhus, Denmark

**Keywords:** Parameter Synthesis, Liveness Properties, IMITATOR

## Abstract

We study semi-algorithms to synthesise the constraints under which a Parametric Timed Automaton satisfies some liveness requirement. The algorithms traverse a possibly infinite parametric zone graph, searching for accepting cycles. We provide new search and pruning algorithms, leading to successful termination for many examples. We demonstrate the success and efficiency of these algorithms on a benchmark. We also illustrate parameter synthesis for the classical Bounded Retransmission Protocol. Finally, we introduce a new notion of completeness in the limit, to investigate if an algorithm enumerates all solutions.

### 1 Introduction

Many critical devices and processes in our society are controlled by software, in which real-time aspects often play a crucial role. Timed Automata (TA [1]) are an important formalism to design and study real-time systems; they extend finite automata with real-valued *clocks*. Their success is based on the decidability of the basic analysis problems of checking *reachability* and *liveness* properties.

Precise timing information is often unknown during the design phase. Therefore, Parametric Timed Automata (PTA [2]) extend TA with *parameters*, representing unknown waiting times, deadlines, network speed, etc. A single PTA represents an infinite class of TA. To facilitate design exploration, *parameter constraint synthesis* aims at a description of all parameter values for which the system meets some requirement. Unfortunately, it is already undecidable to check if a PTA admits a parameter valuation for which a bad state can be reached [2,3].

In this paper, we study the parameter constraint synthesis problem for liveness properties of the full class of PTA. In particular, the goal is to compute the parameter valuations for which a Parametric Timed Büchi Automaton has a non-empty language. Note that this allows handling requirements in LTL and MITL [24]. We represent the solution concisely as a disjunction of conjunctions

*This work is partially supported by projects CNRS-INS2I TrAVAIL, IFD SECReTS and ANR-NRF ProMiS (ANR-19-CE25-0015).

of linear inequalities between the parameters (a set of convex polyhedra).

We will consider semi-algorithms that operate on the so-called parametric zone graph (PZG), where a parametric zone is a conjunction of linear inequalities over clock and parameter values. These semi-algorithms may not terminate since the PZG can be infinite. However, even in that case, we are interested in the soundness and completeness of the set of all enumerated solutions.

Our contributions to the parameter constraint synthesis for liveness of PTA are: 1) A definition of soundness and completeness for non-terminating algorithms. 2) A new synthesis algorithm, using bounded search with iterative deepening; this is the first algorithm that enumerates all accepting cycles in the possibly infinite PZG, in contrast to previous NDFS-based algorithms [25]. 3) An experimental benchmark, comparing the successful termination and runtime efficiency of all algorithms. 4) A case study on the Bounded Retransmission Protocol.

**Related Work.** Decidability for (subclasses of) PTA has been extensively studied [2,19,3]. We study the emptiness and related synthesis problem for *Parametric Timed Büchi Automata* with unrestricted use of rational parameters and real-valued clocks. In this general case, the model checking problem is undecidable [2] and therefore exact synthesis is out of reach (in contrast to the setting with bounded integers [20,11]). Decidability of *liveness* properties for a subclass of PTA, where the occurrence of parameters is restricted, is discussed in [8].

Our approach inherits basic techniques from Timed Automata, in particular the zone graph. For TA, the zone graph is finite after LU-abstraction [27,23,17]. Another technique prunes states that are subsumed by larger states. Subsumption must be applied with care, in order to preserve liveness properties [22,18].

Previous semi-algorithms were based on Nested Depth-First Search (NDFS). They search the (possibly infinite) parametric zone graph (PZG) for accepting cycles. Their zones are projected onto the parameters and accumulated into the global constraint. The basic *cumulative* algorithm [11] prunes states whose projected zone is already included in the accumulated constraint. The cumulative algorithm was extended with *subsumption* and *layering* for PTA [25]. The problem with all NDFS-based algorithms is that the computation can diverge in one branch, missing solutions for accepting cycles in other branches forever.

Our main improvement is a *bounded* approach, which can be combined with breadth- and depth-first search. We check for accepting cycles up to a certain bound, and keep increasing the bound to achieve completeness in the limit. Eventually, this will enumerate all parametric constraints corresponding to all accepting cycles in the PZG. Sometimes, the combination of bounded search and subsumption can even identify infinite paths that do not form a cycle, but this is not guaranteed. A previous proposal for Bounded Model Checking for PTA [21] considers the region graph and has not been implemented. We will provide several small illustrative examples inspired by the invited talk [26].

To evaluate our algorithms, we implemented them in the IMITATOR toolset [6], extending its functionality from reachability to liveness properties. This way, we can reuse its PTA benchmark [4]. We also reimplemented the algorithms of [11,25] in a single NDFS framework. We illustrate our method on the Bounded

Retransmission Protocol (BRP). We synthesize parameter constraints for liveness properties of BRP for the first time. Our constraints are more liberal than the constraints reported in previous work [14,19].

### 2 PTA, Parametric Zone Graphs and Accepted Runs

Let $X$ be a set of real-valued clocks (*e.g.* $x, y$) and let $P$ be a set of rational parameters (*e.g.* $p, q$). A linear term over parameters (*plt*) is an expression of the form $\sum_i \alpha_i p_i + \beta$, where $p_i \in P$, and coefficients $\alpha_i, \beta \in \mathbb{Q}$. A (diagonal) inequality is of the form $x_1 - x_2 \bowtie plt$, with $x_i \in X \cup \{0\}$ and $\bowtie \in \{<, \leq, =, \geq, >\}$. Examples are $x - y \leq 2p + q$, $x > q - 1$ and $2 \leq p$. A (convex) constraint (or zone $Z$) is a conjunction of inequalities. We write $\mathcal{C}$ for the set of zones.

We define a PTA $\mathcal{A} = (L, \ell_0, F, I, E)$, where $L$ is a finite set of locations, $\ell_0 \in L$ is the initial location and $F \subseteq L$ is the set of accepting locations. $I : L \to \mathcal{C}$ denotes an invariant for each location and $E$ is a set of transitions of the form $(\ell, g, R, \ell')$, with source $\ell \in L$, target $\ell' \in L$, guard $g \in \mathcal{C}$ and clock reset $R \subseteq X$.

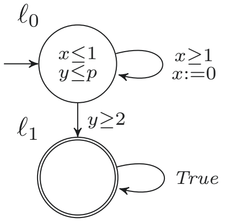

**Fig. 1.** PTA $\mathcal{A}_1$

The concrete semantics of a PTA is defined in terms of valuations. A parameter valuation is a function $v : P \to \mathbb{Q}_{\geq 0}$ and a clock valuation is a function $w : X \to \mathbb{R}_{\geq 0}$. Let $d \in \mathbb{R}_{\geq 0}$ be a delay, then we define the clock valuation $w + d$ such that $(w + d)(x) := w(x) + d$. Let $R \subseteq X$ be a clock reset, then we define the clock valuation $w[R](x) := 0$ if $x \in R$ and $w(x)$ otherwise. We write $\mathbf{0}$ for the clock valuation s.t. $\forall x \in X : \mathbf{0}(x) = 0$. We extend parameter valuations to linear terms. We write $v, w \models (x_i - x_j \bowtie plt)$ iff $w(x_i) - w(x_j) \bowtie v(plt)$, and $v, w \models Z$ iff $v, w \models e$ for all inequalities $e$ in $Z$.

Given a parameter valuation $v$, we write $v(\mathcal{A})$ for the timed automaton obtained by replacing all parameters $p$ in invariants and guards by $v(p)$. The concrete semantics of a PTA $\mathcal{A}$ is derived from the TA $v(\mathcal{A})$, and defined as a timed transition system with states $(\ell, w)$, initial state $(\ell_0, \mathbf{0})$ (we assume that $\mathbf{0} \models I(\ell_0)$), and transitions $\to = \xrightarrow{d} \cdot \xrightarrow{e}$, where continuous time delay ($\xrightarrow{d}$) and discrete transitions ($\xrightarrow{e}$) are defined as

- If $d \in \mathbb{R}_{\geq 0}$ and $w + d \models I(\ell)$, then $(\ell, w) \xrightarrow{d} (\ell, w + d)$.
- If $e = (\ell, g, R, \ell') \in E$ and $w \models g$ and $w[R] \models I(\ell')$ then $(\ell, w) \xrightarrow{e} (\ell', w[R])$.

An infinite run $(\ell_0, w_0) \to (\ell_1, w_1) \to \cdots$ is *accepted* if it passes through an accepting location infinitely often, *i.e.* the set $\{i \mid \ell_i \in F\}$ is infinite. We ignore the problem of Zeno runs, which can be avoided by a syntactic transformation [9].

*Example 1.* The PTA $\mathcal{A}_1$ in Fig. 1 has locations $\{\ell_0, \ell_1\}$, clocks $\{x, y\}$ and parameter $p$. Only $\ell_1$ is accepting. The initial location $\ell_0$ has an invariant consisting of two inequalities. Its self-loop is enabled if $x \geq 1$ and it resets clock $x$. Note that clock $y$ is never reset. For $p = 2.5$, we have the following example run:

$$\big(\ell_0, (0,0)\big) \xrightarrow{1} \big((\ell_0, (1,1)\big) \to \big((\ell_0, (0,1)\big) \xrightarrow{1} \big((\ell_0, (1,2)\big) \to \big((\ell_1, (1,2)\big).$$

Note that the accepting location $\ell_1$ would not be reachable for $p < 2$. On the other hand, for all $p \geq 2$, there exists an infinite accepted run through $\ell_1$.

We will now recall from [5,20] the parametric zone graph (PZG), providing an abstract semantics to a PTA. A single PZG treats all parameter valuations symbolically. Also, the PZG avoids the uncountably infinite timed transition system. The PZG can still be (countably) infinite.

We first define some operations on zones, in terms of their valuations. It is well known that convex polyhedra are closed under these operations, and our implementation in IMITATOR uses the Parma Polyhedra Library [10].

– Time elapse: $Z^{\nearrow}$ corresponds to $\big\{(v, w+d) \mid d \in \mathbb{R}_{\geq 0} \wedge v, w \models Z\big\}$.
– Clock reset: $Z[R]$ corresponds to $\big\{(v, w[R]) \mid v, w \models Z\big\}$.

The PZG is a transition system where each abstract state consists of a location and a non-empty zone. The PZG of $\mathcal{A} = (L, \ell_0, F, I, E)$ is $(S, s_0, \Rightarrow, A)$, with $S \subseteq L \times \mathcal{C}$, initial state $s_0 = (\ell_0, (\bigwedge_{x \in X} x = 0)^{\nearrow} \cap I(\ell_0))$, and accepting states $A = \{(\ell, Z) \mid \ell \in F\}$. A transition step $(\ell, Z) \Rightarrow (\ell', Z')$ exists if for some $(\ell, g, R, \ell') \in E$ we have $Z' = ((Z \cap g)[R] \cap I(\ell'))^{\nearrow} \cap I(\ell') \neq \emptyset$. We write $\Rightarrow^+$ $(\Rightarrow^*)$ for the transitive (reflexive) closure of $\Rightarrow$.

*Example 2.* The PZG of $\mathcal{A}_1$ from Ex. 1 is shown in Fig. 2; it extends infinitely to the right. We use that $(x = 0 \wedge y = 0)^{\nearrow} = (y - x = 0)$. The loop on $\ell_0$ can only be executed when $x = 1$, and it resets $x := 0$, while $y$ is never reset. So after $n$ executions of the loop, $y - x = n$. These $n$ steps are only possible if $p \geq n$.

The PZG obeys two important properties (Prop. 1 and 2). First, the parametric constraint can only decrease along the transitions in the PZG. Second, a state simulates the behaviour of any state that it subsumes. We first define these notions. We write $Z \subseteq Z'$ iff $v, w \models Z$ implies $v, w \models Z'$.

– Parametric constraint: $(\ell, Z){\downarrow_P}$ corresponds to $\{v \mid \exists w. v, w \models Z\}$.
– Subsumption: $(\ell, Z) \sqsubseteq (\ell', Z')$ iff $\ell = \ell'$ and $Z \subseteq Z'$.

**Proposition 1 ([25]).** *If $s_1 \Rightarrow s_2$ then $s_2{\downarrow_P} \subseteq s_1{\downarrow_P}$.*

**Proposition 2 ([25]).** *If $s_1 \Rightarrow s_2$ and $s_1 \sqsubseteq s_1'$ then for some $s_2'$, $s_1' \Rightarrow s_2'$ and $s_2 \sqsubseteq s_2'$.*

*Example 3.* The first $\ell_1$ state in Fig. 2 shows that there is an infinite loop when $p \geq 2$. By Prop. 1, the parametric zone of all states following the dashed red edge are contained in $p \geq 2$. So we can prune the PZG at the dashed red arrow, since no new parameter valuations will be found.

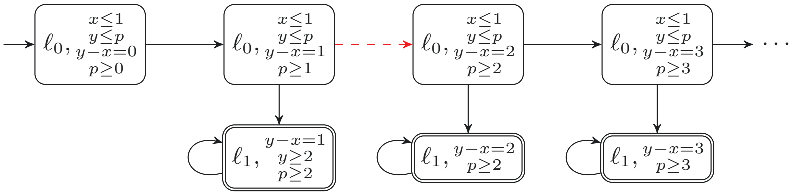

**Fig. 2.** PZG of the PTA $\mathcal{A}_1$ from Fig. 1

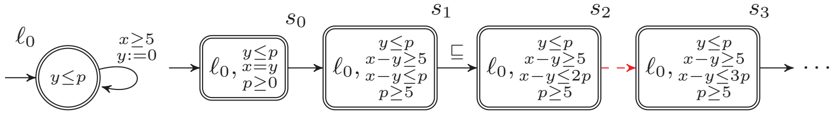

(a) PTA $\mathcal{A}_2$ (b) Its PZG with an infinite accepted run, but no loop

**Fig. 3.** PTA $\mathcal{A}_2$ with the corresponding PZG

*Example 4.* Fig. 3 shows PTA $\mathcal{A}_2$ and its infinite PZG. The transition can only become enabled when $p \geq 5$. Each transition must happen within the following $p$ time units, so after $n > 0$ iterations, $5 \leq x - y \leq n \times p$. Note that $s_1 \Rightarrow s_2$ and $s_1 \sqsubseteq s_2$. By Prop. 2, for some $s'$, $s_2 \Rightarrow s'$ and $s_2 \sqsubseteq s'$. Repeating the argument, we can construct an infinite trace. So, although the PZG has no cycle, the presence of an infinite path can be deduced even if we prune the PZG at the dashed edge.

### 3 Sound and Complete Liveness Parameter Synthesis

Given a PTA $\mathcal{A}$, we aim at synthesising the parameter valuations $v$ for which the TA $v(\mathcal{A})$ contains an infinite accepted run. Our algorithms operate by searching the PZG $(S, s_0, \Rightarrow, A)$ for accepting "lassos" or, as in Ex. 4, 6 and 7, even for accepting "spirals". We write $\Rightarrow^+$ ($\Rightarrow^*$) for the transitive (reflexive) closure of $\Rightarrow$. An accepting lasso on $s_1$ consists of two finite paths $s_0 \Rightarrow^* s_1 \Rightarrow^+ s_1$, such that $s_1 \in A$. More generally, an accepting spiral on $s_1$ consists of two finite paths $s_0 \Rightarrow^* s_1 \Rightarrow^+ s_2$, with $s_1 \in A$ and $s_1 \sqsubseteq s_2$.

**Proposition 3.** *If the PZG of PTA $\mathcal{A}$ contains an accepting spiral on $s_1$, then for all $v \in s_1{\downarrow_P}$, $v(\mathcal{A})$ contains an (infinite) accepted run.*

*Proof.* Assume $s_0 \Rightarrow^* s_1 \Rightarrow^+ s_2$ with $s_1 \in A$ and $s_1 \sqsubseteq s_2$. Note that $s_2 \in A$, since $\sqsubseteq$ only holds between states with the same location. Then by monotonicity, $s_1{\downarrow_P} \sqsubseteq s_2{\downarrow_P}$ and by Prop. 1, $s_2{\downarrow_P} \sqsubseteq s_1{\downarrow_P}$, so $s_1{\downarrow_P} = s_2{\downarrow_P}$. By Prop. 2, there exists some $s_3$ such that $s_2 \Rightarrow s_3$ and $s_2 \sqsubseteq s_3$. We can repeat this to construct an infinite accepted run from $s_1$, with the constant parametric constraint $s_1{\downarrow_P}$. The states from $s_0 \Rightarrow^* s_1$ have an even larger constraint (Prop. 1). By the correspondence between runs in the PTA and runs in the PZG, we obtain an infinite accepted run in $v(\mathcal{A})$ for every $v \models s_1{\downarrow_P}$. □

The reverse of Prop. 3 is not true. An infinite PZG could contain an infinite path that does not form a lasso (or even a spiral). Such an infinite path in the PZG may or may not correspond to a concrete TA run.

*Example 5.* The situation of $\mathcal{A}_3$ in Fig. 4 is quite different from Ex. 4. The PZG of $\mathcal{A}_3$ has an infinite path $(\ell_0, Z_i)$, where $Z_i$ contains the invariant $x \leq 1 \wedge y \leq p$ and the additional constraints $y - x = i \wedge p \geq i$. Note that at most $p$ transitions can happen in $\mathcal{A}_3$, since we cannot wait longer when $y \geq p$. So $v(\mathcal{A}_3)$ has only finite runs for any $v$. We call this infinite path infeasible, since $\cap_i(Z_i{\downarrow_P}) = \emptyset$.

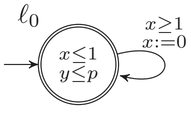

**Fig. 4.** PTA $\mathcal{A}_3$.

#### 3.1 Soundness and Completeness

In contrast to TA, where both reachability and liveness properties are decidable [1], it is well-known that even reachability-emptiness for PTA is undecidable [2,3]. So in particular, we cannot expect a terminating, sound and complete algorithm for liveness synthesis. Instead, our algorithms are *semi-algorithms*, which enumerate a number of aggregate solutions, but may not terminate. Each aggregate solution will be presented as a convex polyhedral constraint on the parameters ("parametric zone").

Such semi-algorithms can either enumerate a finite number of aggregate solutions (after which they could terminate or diverge), or enumerate an infinite number of aggregates (and hence never terminate). Fig. 5 shows an example where the set of solutions, $p \in \{1, 2, 3, \ldots\}$, is not equivalent to a finite disjunction of convex polyhedra, so no terminating algorithm can enumerate all aggregate solutions.[1]

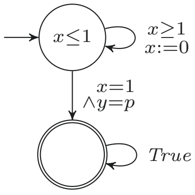

**Fig. 5.** PTA $\mathcal{A}_4$

In the rest of this section, we introduce and discuss various soundness and completeness requirements for semi-algorithms. Assume that the algorithm is run on an input PTA $\mathcal{A}$ and let *Sol* be the set of all solutions, *i.e.* $Sol = \{v \mid v(\mathcal{A})$ has an accepted run$\}$. Assume that the algorithm enumerates a finite or infinite collection of aggregate solutions, in the form of parametric zones $Z_i$.

*Partial correctness:* This traditional correctness criterion requires that if the algorithm terminates, then $\bigcup_i Z_i = Sol$, *i.e.* the finite output characterizes exactly all correct parameter valuations.

*Soundness:* This criterion also provides some guarantee when the algorithm diverges. It requires that all enumerated solutions are correct, *i.e.* $\bigcup_i Z_i \subseteq Sol$.

*Completeness:* We call a semi-algorithm *complete* if it enumerates all solutions, *i.e.* $Sol \subseteq \bigcup_i Z_i$. Enumerating $p = 1$, $p = 2$, $\ldots$ is complete for $\mathcal{A}_4$.

Note that for reachability, a simple Breadth-First Search (BFS) over the PZG would yield a sound and complete (but not always terminating) semi-algorithm. For liveness, this is insufficient: the algorithm would miss infinite paths that do not form a cycle. Still, the following trivial semi-algorithm, EnumQ, would be sound and complete: "Enumerate all rational parameter valuations $v$, decide if $v(\mathcal{A})$ has an accepting loop [1] and, if so, emit $\{v\}$." Although it is sound and complete, this algorithm is quite unsatisfactory, since it will never terminate, and it will never aggregate solutions in larger polyhedra. To distinguish PZG-based algorithms, we need a weaker form of completeness.

*Completeness for symbolic lassos:* A semi-algorithm is *complete for symbolic lassos* if it enumerates all parameter valuations leading to accepting lassos in the PZG, *i.e.* $\bigcup_i Z_i$ contains $s{\downarrow_P}$, when the PZG contains an accepting lasso on $s$.

Completeness for symbolic lassos is weaker than completeness, since it may miss parameter valuations $v$ for which $v(\mathcal{A})$ has an accepted run, but this only happens when the PZG has an infinite path that does not end in a cycle.

[1] It is not even obvious that $\cap_i (Z_i{\downarrow_P})$ can be represented by a finite conjunction.

## 4 Semi-Algorithms for Liveness Parameter Synthesis

In this section, we discuss three semi-algorithms for liveness parameter synthesis. In Sec. 4.1, we discuss the previous approach [11,25], based on Nested Depth-First Search (NDFS). All NDFS-based variants turn out to be incomplete for symbolic lassos. In Sec. 4.2, we introduce a simple algorithm based on Breadth-First Search (BFS), which analyses the Strongly Connected Components (SCC) at each new level. We show that the BFS-based algorithm is complete for symbolic lassos. Finally, Sec. 4.3 introduces our new Bounded Synthesis with Iterative Deepening (BSID) algorithm. BSID is also complete for symbolic lassos, and it is compatible with all NDFS enhancements.

### 4.1 Nested Depth-First Search with Enhancements

The NDFS algorithm (Alg. 1) is run on the PZG, with initial state $s_0$, accepting states $A$, and NEXT-STATE($s$) enumerating the $\Rightarrow$-successors. We first explain basic NDFS [13], cf. the uncoloured parts of Alg. 1. The goal of the outer *blue search* (ll.4–13) is to visit all states in DFS order, and just before backtracking, call the red search on all accepting states (l.12). Note that states on the DFS stack are cyan (l.6), and states that are handled completely are blue (l.13). The goal of the inner *red search* (ll.14–21) is to detect if there is an accepting cycle. It colours visited states red (l.16), to ensure that states are visited at most once. It reports an accepting cycle (l.20) when a cyan state is encountered.

*Cumulative pruning* (pink) [11,25]. For synthesis, we collect the *Constraints* that lead to accepting cycles (l.20). We prune the search when the parametric constraint of some state is included in *Constraints* (l.5,15). This is justified by Prop. 1, since all successors of the pruned state will have an even smaller parametric constraint. Prop. 1 also implies that all states on a cycle have the same parametric constraint. So we also prune the red search, by restricting the search for a cycle to the current parametric constraint (l.18).

*Subsumption* (grey) [22,25]. This pruning strategy takes advantage of the subsumption relation between states. The accepting lassos reachable from red states $s$ are already included in *Constraints*. By Prop. 3, any lasso on state $t \sqsubseteq t'$ can be simulated by $t'$. Hence, we immediately prune the search when we encounter a state $t \sqsubseteq Red$, *i.e.* $\exists t'.\, t \sqsubseteq t' \in Red$ (l.11,21). We exploit the subsumption structure once more: if $t \sqsupseteq Cyan$, *i.e.* $\exists t'.\, t \sqsupseteq t' \in Cyan$ (l.19), we have found an accepting spiral, which implies there is an accepted run, Prop. 3.

*Lookahead* (yellow). The *lookahead* strategy is new (in this context) and allows for early detection of accepting cycles in *dfsBlue*. It looks for a transition to a *cyan* state (l.7), which is on the DFS stack. If the source or target of this transition is accepting, then the cycle is accepting as well and reported at l.8.

*Accepting First* (blue). This is a new strategy, aimed at increasing the chance of finding an accepting cycle early in the search, to promote more pruning. It simply works by picking accepting successors before their siblings at l.10,17.

**Alg. 1** Collecting NDFS with strategies:
cumulative pruning | subsumption | lookahead | accepting first

```
procedure NDFS
    Cyan := Blue := Red := ∅ ;  Constraints := ∅
    dfsBlue(s0)

procedure dfsBlue(s)
    if s↓P ⊆ Constraints then Blue := Blue ∪ {s} ; return
    Cyan := Cyan ∪ {s}
    if ∃s′ ∈ NEXT-STATE(s) ∩ Cyan : (s ∈ A ∨ s′ ∈ A) then
        Constraints := Constraints ∪ {s′↓P}            ▷ Report cycle at state s’
    else
        for all t ∈ REORDERED-NEXT-STATE(s) do
            if t ∉ Blue ∪ Cyan ∧ t ⋢ Red then dfsBlue(t)
        if s ∈ A then dfsRed(s)
    Blue := Blue ∪ {s}; Cyan := Cyan \ {s}

procedure dfsRed(s)
    if s↓P ⊈ Constraints then
        Red := Red ∪ {s}
        for all t ∈ REORDERED-NEXT-STATE(s) do
            if t↓P= s↓P then
                if Cyan ⊑ t then
                    Constraints := Constraints ∪ t↓P     ▷ Report cycle at state t
                else if t ⋢ Red then dfsRed(t)
```

*Layering* (not shown here) [25]. The layering strategy gives priority to states with large parametric constraints, since these potentially prune many other states. To this end, successors in the next parametric layer are delayed, which is sound, since every cycle must lie entirely in the same parametric layer (Prop. 1).

**Proposition 4.** *All mentioned NDFS variants are sound and partially correct.*

*Proof.* Partial correctness is shown in [25]. Soundness follows from Prop. 3, since all collected constraints correspond to accepting spirals. □

*Example 6.* None of the mentioned NDFS is complete for symbolic lassos. Consider $\mathcal{A}_5$ in Fig. 6. Its PZG extends Fig. 3(b) with a transition from all states to one additional accepting state with self-loop, $s = (\ell_1, p + x \geq y \geq 6 + x)$, where $s{\downarrow_P} = (p \geq 6)$. All NDFS variants (including all combinations of cumulative pruning, subsumption, lookahead, accept-first, and layering) allow the execution that diverges on the infinite $p \geq 5$ path, so they will never detect the accepting cycle on $p \geq 6$.

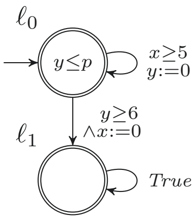

**Fig. 6.** PTA $\mathcal{A}_5$

#### 4.2 Breadth-First Search

We now describe a BFS-based synthesis algorithm for accepting cycle detection. As in Alg. 1, our BFS algorithm maintains a parameter constraint *Constraints*, initially empty. The algorithm basically explores the newly computed symbolic states in a breadth-first search manner, *i.e.* by iteratively computing all siblings at a given depth level, before computing their own children states. Then, whenever one of these new states is *identical* to a state already present in the state space, a cycle may exist. In this case, we run an SCC-detection algorithm (inspired by Tarjan) and, if there is indeed a cycle, we add the cycle parameter constraint to the result *Constraints*. Remember that, from Prop. 1, all states in such a cycle have the same parametric constraint.

Note that, in contrast to the algorithms in Sec. 4.1 and 4.3, we have to use state *equality*, since using unrestricted subsumption could introduce spurious cycles (cf. examples in [22]). However, we do use *cumulative pruning*, as in Sec. 4.1: whenever the parametric constraint of a new state $s$ is included in the current result *Constraints* (*i.e.* $s{\downarrow_P} \subseteq \mathit{Constraints}$), we discard it, as no potential loop starting from this state, or from its successors, can improve *Constraints* anyhow.

In contrast to the NDFS-based algorithms in Sec. 4.1, our BFS algorithm is complete for symbolic lassos, since every lasso will appear at some level, and the SCC algorithm will eventually detect it.

**Proposition 5.** *The BFS+SCC algorithm is sound, partially correct, and complete for symbolic lassos.*

#### 4.3 Bounded Synthesis with Iterative Deepening

One way to enforce termination is to explore the PZG up to a given depth (Bounded Synthesis). However, this could make the result incomplete. Therefore, as long as there are unexplored states, the bound should be increased (Iterative Deepening), to synthesize parameter valuations for deeper accepting cycles.

Alg. 2 presents this procedure, called BSID. Although all strategies in Sec. 4.1 are compatible with this approach, only cumulative pruning and subsumption are shown in the algorithm. It repeatedly explores the PZG from an initial depth *depthinit*, incrementing the depth by *depthstep* at each iteration (l.8). The termination criterion is that the current exploration did terminate without reaching its current depth (l.7). In this case, the result is complete. Both *dfsBlue* and *dfsRed* do not go beyond the current exploration depth (at l.10,20).

To avoid some duplicate work at different iterations, the set of blue states is split using two colours: *Green states* have a descendent not completely processed due to the depth limit, and should thus be considered in further iterations; *Blue states* are those whose children have already been completely explored and thus should not be considered anymore. Hence, at the beginning of an iteration, all colours but blue are reset (l.5). States are coloured green when they are at the depth limit (l.10) or if they have a green successor (l.16). Note that *dfsBlue* is not called for blue states at l.14, but it may be called for states that have been coloured green at the previous iteration but have been uncoloured.

**Proposition 6.** *The BSID algorithm is sound, partially correct, and complete for symbolic lassos.*

*Proof.* Soundness follows from Prop. 3, since every collected constraint corresponds to an accepting spiral. Completeness for symbolic lassos follows, since every accepting cycle in the PZG is entirely present at some depth. When NDFS is run beyond that depth, it will report the constraint leading to that cycle. Partial correctness follows, since the algorithm only terminates if the last run did not reach the depth-bound, in which case the PZG is searched exhaustively. □

*Example 7.* On both $\mathcal{A}_2$ (Fig. 3, Ex. 4) and $\mathcal{A}_5$ (Fig. 6, Ex. 6), BSID will correctly report $p \geq 5$ and then terminate; for $\mathcal{A}_5$ it may first report $p \geq 6$, depending on the search order. It is actually the combination of *bounded* synthesis and *subsumption* that makes the algorithm complete for this example. The bound ensures that NDFS is run after the first iteration, and subsumption ensures that an accepting spiral is found as explained in Ex. 4. At this point, the constraint $p \geq 5$ is discovered, which prunes the rest of the PZG, ensuring termination.

**Alg. 2** Iterative deepening NDFS with cumulative constraint pruning and subsumption

```
procedure IterativeCollectNDFSsub(depthinit, depthstep)
    Cyan := Blue := Red := Green := ∅ ; Constraints := ∅
    depth := depthinit; again := true
    while again do
        Cyan := Red := Green := ∅ ; depthreached := false
        dfsBlue(s_0, 0)
        if ¬depthreached then again := false
        if again then depth := depth + depthstep
procedure dfsBlue(s, d_s)
    if d_s ≥ depth then depthreached := true ; Green := Green ∪ {s} ; return
    if s↓_P ⊆ Constraints then Blue := Blue ∪ {s} ; return
    Cyan := Cyan ∪ {s}
    for all t ∈ next-state(s) do
        if t ∉ Blue ∪ Green ∪ Cyan ∧ t ⋢ Red then dfsBlue(t, d_s+1)
    if s ∈ A then dfsRed(s, d_s)
    if ∃s' ∈ Green ∩ next-state(s) then Green := Green ∪ {s}
    else Blue := Blue ∪ {s}
    Cyan := Cyan \ {s}
procedure dfsRed(s, d_s)
    if d_s < depth ∧ s↓_P ⊈ Constraints then
        Red := Red ∪ {s}
        for all t ∈ next-state(s) do
            if t↓_P = s↓_P then
                if Cyan ⊑ t then
                    Constraints := Constraints ∪ t↓_P        ▷ Report cycle at state t
                else if t ⋢ Red then dfsRed(t, d_s+1)
```

### 5 Experimental Evaluation

We conducted some experiments, to compare all algorithms on the number of cases they can solve and on their efficiency. In order to compare cases in which an algorithm does not terminate, we also counted the number of reported cycles.

To this end, we implemented our new algorithms BFS and BSID in IMITATOR 3,[2] and we also reimplemented all NDFS-based algorithms [11,25] in a unified DFS framework. We ran all algorithms on a benchmark, distributed with IMITATOR [4] and also used in [25]. The size of the benchmarks is shown in Tab. 1 (columns L,X,P). We used a timeout of 120 s.[3]

In Tab. 1, we compare some combinations of NDFS enhancements (Sec. 4.1), extending the baseline (*cumulative pruning*). The results show that *subsumption* alone performs worst, while *lookahead* solves more cases, *e.g.* ll.3–6 of Tab. 1. Interestingly, adding our new *accepting first* strategy succeeds to find cycles (l.12) that are missed by all other strategies. Finally, adding the *layering* approach leads to success in most cases and provides the fastest results on average, but it finds no accepting cycles at all for five cases where others found some.

Tab. 2 compares the new algorithms BFS (Sec. 4.2) and BSID (Sec. 4.3), including all enhancements (except layering) under various depth settings. BSID is generally faster than BFS, in particular with an iterative depth-step of 5. The performance of BFS is closest to BSID with depth-step 1. The first two columns evaluate the effectiveness of using the green colour (ng = `-no-green`). Without green, no information from previous iterations is reused. Avoiding recomputation is faster, leading to a deeper exploration within the time limit (*e.g.* on l.2).

Comparing both tables, we notice that for ll.15–17 NDFS synthesised some parameter values that are missed by BSID and BFS. BSID is generally faster than its NDFS counterpart A+L+Sub, but NDFS with layering is even faster.

### 6 Case Study: the Bounded Retransmission Protocol

The *Bounded Retransmission Protocol* (BRP) has been analysed in [16,14,19], but we now *synthesise* the most liberal parameter constraints to obtain some reachability and liveness guarantees. For reachability, these constraints are more liberal than proposed in previous work. Synthesising parameter constraints for liveness properties is new, and our new algorithms were required to achieve this.

Our starting point is the PTA model from [14]. Each session starts with a transmission request S_in and is terminated by an indication S_ok, S_nok or S_dk ("don't know"). The BRP is regulated by clocks, with some timing parameters: TD is the delay in the communication channel, TS and TR indicate the time that the sender (receiver) should wait. Finally, SYNC models the waiting time in case sender and receiver get out of sync. The maximum number of retransmissions is a discrete parameter, which we fixed in most experiments to MAX = 2.

[2] Algorithms are integrated in IMITATOR v.3. The artifact is at doi.org/10.5281/zenodo.4115919 and can be run at: imitator.lipn.univ-paris13.fr/artifact.

[3] The experiment ran on a DELL PowerEdge FC640, 2 processors (Intel Xeon Silver 4114 @ 2.20 GHz), Debian GNU/Linux 10, 187.50 GiB memory.

| | Model | L | X | P | Subsumption | | | | | Lookahead | | | | | Accept+Look | | | | | A + L + Sub | | | | | A + L + Sub + layers | | | | |
|---|---|---|---|---|---|---|---|---|---|---|---|---|---|---|---|---|---|---|---|---|---|---|---|---|---|---|---|---|---|
| | | | | | d | m | c | s | t | d | m | c | s | t | d | m | c | s | t | d | m | c | s | t | d | m | c | s | t |
| 1 | BRP | 22 | 7 | 2 | 32 | 18 | 17 | 8370 | TO | 32 | 15 | 17 | 8336 | TO | 32 | 14 | 17 | 8340 | TO | 32 | 14 | 17 | 8350 | TO | 31 | 16 | 10 | 4666 | TO |
| 2 | coffee | 4 | 2 | 3 | 3580 | — | 0 | 3581 | TO | 3590 | — | 0 | 3591 | TO | 3591 | — | 0 | 3592 | TO | 3581 | — | 0 | 3582 | TO | 3545 | 4 | 1 | 3550 | TO |
| 3 | critical-region | 20 | 2 | 2 | 10980 | — | 0 | 10981 | TO | 4 | 3 | 2 | 7 | 0.013 | 4 | 3 | 2 | 6 | 0.015 | 4 | 3 | 2 | 6 | 0.013 | 3 | 3 | 1 | 4 | 0.012 |
| 4 | critical-region4 | 38 | 4 | 2 | 11564 | — | 0 | 11565 | TO | 10 | 7 | 2 | 17 | 0.042 | 10 | 7 | 2 | 16 | 0.044 | 10 | 7 | 2 | 16 | 0.043 | 7 | 7 | 1 | 8 | 0.026 |
| 5 | F3 | 18 | 3 | 0 | 7607 | — | 0 | 7608 | TO | 0 | 0 | 1 | 1 | 0.009 | 0 | 0 | 1 | 1 | 0.007 | 0 | 0 | 1 | 1 | 0.009 | 0 | 0 | 1 | 1 | 0.007 |
| 6 | F4 | 23 | 4 | 2 | 6260 | — | 0 | 6261 | TO | 0 | 0 | 1 | 1 | 0.011 | 0 | 0 | 1 | 1 | 0.009 | 0 | 0 | 1 | 1 | 0.009 | 0 | 0 | 1 | 1 | 0.012 |
| 7 | FDDI4 | 34 | 13 | 2 | 90 | 36 | 2 | 742 | 1.960 | 90 | 32 | 2 | 690 | 1.695 | 90 | 32 | 2 | 690 | 1.692 | 90 | 32 | 2 | 690 | 1.696 | 110 | 101 | 1 | 660 | 1.718 |
| 8 | FischerAHV93 | 13 | 2 | 4 | 15 | 4 | 2 | 24 | 0.022 | 4 | 3 | 1 | 11 | 0.013 | 4 | 3 | 1 | 11 | 0.013 | 4 | 3 | 1 | 11 | 0.012 | 4 | 3 | 1 | 11 | 0.013 |
| 9 | flipflop | 49 | 5 | 2 | 7 | 5 | 6 | 20 | 0.024 | 7 | 5 | 6 | 18 | 0.022 | 7 | 5 | 6 | 18 | 0.022 | 7 | 5 | 6 | 18 | 0.023 | 7 | 7 | 1 | 8 | 0.014 |
| 10 | fmtv1A1-v2 | 15 | 3 | 3 | 30 | 13 | 75 | 4229 | TO | 30 | 13 | 75 | 4235 | TO | 30 | 13 | 66 | 4538 | TO | 30 | 13 | 66 | 4539 | TO | 29 | 13 | 55 | 5929 | 67.651 |
| 11 | fmtv1A3-v2 | 15 | 3 | 3 | 40 | 26 | 116 | 4949 | TO | 40 | 26 | 116 | 4975 | TO | 40 | 26 | 108 | 5065 | TO | 40 | 26 | 108 | 5041 | TO | 45 | — | 0 | 10898 | TO |
| 12 | JLR-TACAS13 | 2 | 2 | 2 | 6443 | — | 0 | 6444 | TO | 6506 | — | 0 | 6507 | TO | 1309 | 1 | 1309 | 2619 | TO | 1308 | 1 | 1308 | 2616 | TO | 6362 | — | 0 | 6363 | TO |
| 13 | lynch | 18 | 2 | 1 | 4 | 3 | 1 | 5 | 0.008 | 3 | 3 | 1 | 4 | 0.009 | 3 | 3 | 1 | 4 | 0.011 | 3 | 3 | 1 | 4 | 0.009 | 3 | 3 | 1 | 4 | 0.012 |
| 14 | lynch5 | 45 | 5 | 1 | 10 | 9 | 1 | 24 | 0.033 | 7 | 7 | 1 | 19 | 0.027 | 7 | 7 | 1 | 19 | 0.028 | 7 | 7 | 1 | 19 | 0.025 | 7 | 7 | 1 | 19 | 0.025 |
| 15 | Pipeline-KP12-2-3 | 14 | 4 | 6 | 544 | 81 | 29 | 3256 | TO | 560 | 36 | 59 | 2227 | TO | 73 | 29 | 80 | 1643 | TO | 73 | 29 | 80 | 1645 | TO | 49 | 32 | 46 | 2146 | TO |
| 16 | Pipeline-KP12-2-5 | 18 | 4 | 6 | 997 | 756 | 10 | 2989 | TO | 110 | 50 | 64 | 2401 | TO | 101 | 39 | 69 | 1711 | TO | 101 | 39 | 69 | 1713 | TO | 69 | 44 | 33 | 2201 | TO |
| 17 | Pipeline-KP12-3-3 | 19 | 5 | 6 | 689 | 448 | 6 | 1263 | TO | 132 | 57 | 23 | 869 | TO | 122 | 50 | 21 | 706 | TO | 122 | 50 | 21 | 707 | TO | 112 | 59 | 4 | 1239 | TO |
| 18 | RCP | 48 | 6 | 5 | 74 | 8 | 12 | 237 | 0.886 | 74 | 8 | 12 | 237 | 0.877 | 74 | 8 | 12 | 237 | 0.871 | 74 | 8 | 12 | 237 | 0.866 | 50 | 50 | 1 | 105 | 0.152 |
| 19 | Sched2.100.0 | 17 | 6 | 2 | 132 | 3 | 19 | 872 | 5.890 | 132 | 3 | 19 | 872 | 5.870 | 132 | 2 | 19 | 869 | 5.842 | 132 | 2 | 19 | 869 | 5.844 | 174 | 20 | 4 | 592 | 2.465 |
| 20 | Sched2.100.2 | 17 | 6 | 2 | 990 | 3 | 21 | 2430 | TO | 990 | 3 | 21 | 2453 | TO | 990 | 2 | 21 | 2453 | TO | 990 | 2 | 21 | 2461 | TO | 3433 | — | 0 | 3838 | TO |
| 21 | Sched2.50.0 | 17 | 6 | 2 | 132 | 7 | 19 | 756 | 4.434 | 132 | 7 | 19 | 756 | 4.398 | 132 | 6 | 19 | 752 | 4.348 | 132 | 6 | 19 | 752 | 4.368 | 242 | 31 | 5 | 636 | 2.853 |
| 22 | Sched2.50.2 | 17 | 6 | 2 | 1559 | 7 | 22 | 3037 | TO | 1561 | 7 | 22 | 3039 | TO | 1563 | 6 | 22 | 3037 | TO | 1567 | 6 | 22 | 3041 | TO | 2737 | — | 0 | 4584 | TO |
| 23 | simop | 46 | 8 | 2 | 2533 | — | 0 | 2534 | TO | 2520 | — | 0 | 2521 | TO | 2520 | — | 0 | 2521 | TO | 2521 | — | 0 | 2522 | TO | 2520 | — | 0 | 2521 | TO |
| 24 | spsmall | 52 | 11 | 2 | 34 | 30 | 142 | 4036 | 17.637 | 34 | 26 | 142 | 3445 | 13.812 | 34 | 25 | 142 | 2634 | 10.952 | 34 | 25 | 142 | 2634 | 10.987 | 34 | 25 | 142 | 2663 | 7.436 |
| 25 | tgcTogether2 | 12 | 3 | 6 | 32 | 13 | 7 | 137 | 0.410 | 18 | 13 | 4 | 79 | TO | 18 | 13 | 4 | 79 | TO | 18 | 13 | 4 | 79 | 0.193 | 14 | 13 | 2 | 47 | 0.060 |
| 26 | WFAS-BBLS15-det | 10 | 4 | 2 | 6682 | 3 | 12 | 6737 | TO | 6749 | 3 | 12 | 6804 | TO | 6643 | 3 | 14 | 6698 | TO | 6576 | 3 | 14 | 6631 | TO | 7048 | — | 0 | 7049 | TO |
| | # terminations | | | | 10 | | | | | 13 | | | | | 13 | | | | | 14 | | | | | 15 | | | | |
| | # fastest | | | | 1 | | | | | 0 | | | | | 3 | | | | | 3 | | | | | 11 | | | | |
| | Avg. Norm. Time | | | | 0.853 | | | | | 0.754 | | | | | 0.743 | | | | | 0.720 | | | | | 0.651 | | | | |

**Table 1.** Comparing various NDFS enhancements. For each model, L denotes the number of locations, X the number of clocks, and P the number of parameters. For each algorithm, column d indicates the actual depth reached, m the minimum depth at which a cycle was found, c the total number of cycles found, s the number of states explored, and t the time spent in the algorithm (discarding parsing the model) in seconds. # terminations indicates the number of benchmarks for which the algorithm terminates, and # fastest how many times it performed best. Finally, we computed for each algorithm the Average Normalised Time over all benchmarks, where we normalised the time w.r.t. the largest time used by any algorithm in Tab. 1 and 2. Timeout values get a normalised time of 1.

| | Model | Depth 5, step 5 | | | | Depth 5, step 5 (ng) | | | | Depth 10, step 5 | | | | Depth 10, step 10 | | | | Depth 0, step 1 | | | | BFS | | | |
|---|---|---|---|---|---|---|---|---|---|---|---|---|---|---|---|---|---|---|---|---|---|---|---|---|---|
| | | d | m | c | t | d | m | c | t | d | m | c | t | d | m | c | t | d | m | c | t | d | m | c | t |
| 1 | BRP | 20 | 12 | 19 | TO | 20 | 12 | 19 | TO | 20 | 12 | 19 | TO | 20 | 12 | 26 | TO | 20 | 12 | 15 | TO | 21 | 13 | 41 | TO |
| 2 | coffee | 2280 | 4 | 1 | TO | 2055 | 4 | 1 | TO | 2273 | 4 | 6 | TO | 2674 | 4 | 6 | TO | 1345 | 4 | 1 | TO | 3664 | 5 | 4 | TO |
| 3 | critical-region | 4 | 3 | 2 | 0.012 | 4 | 3 | 2 | 0.013 | 4 | 3 | 2 | 0.015 | 4 | 3 | 2 | 0.014 | 2 | 2 | 1 | 0.015 | 5 | 3 | 2 | 0.023 |
| 4 | critical-region4 | 5 | 5 | 1 | 0.141 | 5 | 5 | 1 | 0.141 | 10 | 7 | 2 | 0.042 | 10 | 7 | 2 | 0.044 | 4 | 4 | 1 | 0.432 | 7 | 5 | 29 | 3.321 |
| 5 | F3 | 0 | 0 | 1 | 0.007 | 0 | 0 | 1 | 0.007 | 0 | 0 | 1 | 0.008 | 0 | 0 | 1 | 0.007 | 0 | 0 | 1 | 0.009 | 3 | 1 | 4 | 0.010 |
| 6 | F4 | 0 | 0 | 1 | 0.009 | 0 | 0 | 1 | 0.011 | 0 | 0 | 1 | 0.010 | 0 | 0 | 1 | 0.009 | 0 | 0 | 1 | 0.011 | 3 | 1 | 5 | 0.014 |
| 7 | FDDI4 | 70 | 32 | 2 | 2.279 | 70 | 32 | 2 | 2.658 | 70 | 32 | 2 | 2.308 | 70 | 32 | 2 | 2.010 | 70 | 32 | 2 | 3.366 | 72 | 33 | 10 | 2.907 |
| 8 | FischerAHV93 | 4 | 3 | 1 | 0.010 | 4 | 3 | 1 | 0.013 | 4 | 3 | 1 | 0.014 | 4 | 3 | 1 | 0.014 | 4 | 3 | 1 | 0.017 | 6 | 1 | 14 | 0.010 |
| 9 | flipflop | 7 | 5 | 6 | 0.022 | 7 | 5 | 6 | 0.023 | 7 | 5 | 6 | 0.022 | 7 | 5 | 6 | 0.021 | 7 | 5 | 6 | 0.026 | 9 | 6 | 8 | 0.024 |
| 10 | fmtv1A1-v2 | 30 | 13 | 51 | 119.408 | 30 | 13 | 51 | TO | 30 | 13 | 51 | 119.010 | 30 | 13 | 60 | TO | 29 | 13 | 45 | 102.716 | 32 | 14 | 321 | 113.084 |
| 11 | fmtv1A3-v2 | 20 | 13 | 21 | TO | 20 | 13 | 21 | TO | 20 | 13 | 21 | TO | 20 | 13 | 22 | TO | 20 | 13 | 10 | TO | 21 | 14 | 101 | TO |
| 12 | JLR-TACAS13 | 2065 | 1 | 2065 | TO | 1490 | 1 | 1490 | TO | 2065 | 1 | 2065 | TO | 2690 | 1 | 2690 | TO | 1030 | 1 | 1030 | TO | 1299 | 2 | 1298 | TO |
| 13 | lynch | 3 | 3 | 1 | 0.008 | 3 | 3 | 1 | 0.011 | 3 | 3 | 1 | 0.013 | 3 | 3 | 1 | 0.010 | 3 | 3 | 1 | 0.012 | 6 | 4 | 1 | 0.013 |
| 14 | lynch5 | 5 | 5 | 1 | 0.100 | 5 | 5 | 1 | 0.094 | 7 | 7 | 1 | 0.025 | 7 | 7 | 1 | 0.028 | 3 | 3 | 1 | 0.120 | 6 | 4 | 1 | 0.437 |
| 15 | Pipeline-KP12-2-3 | 15 | — | 0 | TO | 15 | — | 0 | TO | 15 | — | 0 | TO | 20 | — | 0 | TO | 13 | — | 0 | TO | 15 | — | 0 | TO |
| 16 | Pipeline-KP12-2-5 | 15 | — | 0 | TO | 15 | — | 0 | TO | 15 | — | 0 | TO | 20 | — | 0 | TO | 15 | — | 0 | TO | 17 | — | 0 | TO |
| 17 | Pipeline-KP12-3-3 | 15 | — | 0 | TO | 15 | — | 0 | TO | 15 | — | 0 | TO | 20 | — | 0 | TO | 13 | — | 0 | TO | 16 | — | 0 | TO |
| 18 | RCP | 10 | 8 | 6 | 0.507 | 10 | 8 | 6 | 0.511 | 10 | 8 | 6 | 0.352 | 10 | 8 | 6 | 0.351 | 10 | 8 | 7 | 0.834 | 12 | 9 | 104 | 1.855 |
| 19 | Sched2.100.0 | 104 | 2 | 10 | 5.709 | 107 | 2 | 10 | 7.044 | 104 | 2 | 10 | 5.715 | 104 | 2 | 10 | 4.322 | 104 | 2 | 9 | 19.068 | 54 | 3 | 56 | 2.861 |
| 20 | Sched2.100.2 | 130 | 2 | 14 | TO | 130 | 2 | 14 | TO | 130 | 2 | 14 | TO | 140 | 2 | 14 | TO | 120 | 2 | 13 | TO | 92 | 3 | 348 | TO |
| 21 | Sched2.50.0 | 104 | 6 | 9 | 4.054 | 107 | 6 | 9 | 5.044 | 104 | 6 | 9 | 4.048 | 104 | 6 | 9 | 3.043 | 104 | 6 | 8 | 13.177 | 36 | 7 | 52 | 1.900 |
| 22 | Sched2.50.2 | 135 | 6 | 12 | TO | 135 | 6 | 12 | TO | 135 | 6 | 12 | TO | 140 | 6 | 12 | TO | 131 | 6 | 11 | TO | 106 | 7 | 278 | TO |
| 23 | simop | 25 | 15 | 53 | TO | 25 | 15 | 53 | TO | 25 | 15 | 53 | TO | 30 | 20 | 59 | TO | 21 | 13 | 44 | TO | 22 | 14 | 304 | TO |
| 24 | spsmall | 34 | 25 | 142 | 9.632 | 34 | 25 | 142 | 9.633 | 34 | 25 | 142 | 9.581 | 34 | 25 | 142 | 11.246 | 34 | 25 | 142 | 11.318 | 36 | 26 | 368 | 12.480 |
| 25 | tgcTogether2 | 15 | 13 | 3 | 0.149 | 15 | 13 | 3 | 0.155 | 15 | 13 | 3 | 0.154 | 18 | 13 | 4 | 0.197 | 14 | 13 | 3 | 0.197 | 16 | 14 | 5 | 0.159 |
| 26 | WFAS-BBLS15-det | 2090 | 3 | 9 | TO | 1465 | 3 | 9 | TO | 2090 | 3 | 12 | TO | 2800 | 3 | 12 | TO | 991 | 3 | 9 | TO | 6748 | 4 | 17 | TO |
| | # terminations | 15 | | | | 14 | | | | 15 | | | | 14 | | | | 15 | | | | 15 | | | |
| | # fastest | 6 | | | | 1 | | | | 3 | | | | 5 | | | | 1 | | | | 3 | | | |
| | Avg. Norm. Time | 0.701 | | | | 0.735 | | | | 0.725 | | | | 0.704 | | | | 0.835 | | | | 0.863 | | | |

**Table 2.** Comparing exploration of BSID (Alg. 2) with different depth settings, using all strategies except layering (A+L+Sub), and BFS (Sec. 4.2). For each algorithm, column d indicates the actual depth reached, m the minimum depth at which a cycle was found, c the total number of cycles found, and t the time spent in the algorithm (discarding parsing the model) in seconds. # terminations indicates the number of benchmarks for which the algorithm terminates, and # fastest how many times it performed best. Finally, we computed for each algorithm the Average Normalised Time over all benchmarks, where we normalised the time w.r.t. the largest time used by any algorithm in Tab. 1 and 2. Timeout values get a normalised time of 1.

#### 6.1 Synthesis for Reachability Properties: deriving sharper bounds

To illustrate synthesis for reachability properties, we first enhance the parametric verification experiments from [14,19] in IMITATOR. The reachability properties are: **(C)** the channels will never be used simultaneously; and **(R)** the receiver gets a correct initial frame in each session. Property **(C)** is formalised as:

```
property := #synth AGnot(loc[channelK] = in_transitK & loc[channelL] = in_transitL)
```

We synthesise the safe parameter constraints for "unreachability" by:[4]

```
imitator -mergeq -comparison inclusion brp_Channels.imi brp_Channels.imiprop
```

IMITATOR derives within 2 s the exact constraint `TS > 2*TD`: The sender should wait (TS) for the round-trip time of a message + acknowledgement (`2*TD`).

Property **(R)** is formalised by adding an error location `FailureR` to the receiver, which should be unreachable. Since we learned the constraint `TS>2*TD` in the previous run, we now include this constraint in the initial condition. Within 1 s, IMITATOR synthesizes the exact constraint for this safety property:

```
imitator -mergeq -comparison inclusion brp_RC.imi brp_RC.imiprop
SYNC + TS >= TR + TD & TS > 2*TD & TR > 4*TS + 3*TD
```

The fact that this can be computed is not surprising, but it *is* surprising that this constraint is more liberal than the one derived in [14,19], which was:

```
SYNC >= TR & TS > 2*TD & TR > 2*MAX*TS + 3*TD
```

One can easily check that, for MAX = 2, their constraint is strictly stronger than ours. *So we found more parameter values for which BRP is correct.* By construction, we found the most liberal constraint for MAX = 2, and we confirmed a similar result for up to MAX = 20. We cannot handle a parametric MAX.

#### 6.2 Liveness: approximations by bounded synthesis

Next, we want to measure the overhead of liveness checking. To this end, we make the failureR location an accepting cycle, and use a liveness property. Note that in this case, the synthesised constraint will indicate the error condition.

```
accepting loc FailureR: invariant True when True goto FailureR;
init := ... & TS > 2 * TD
property := #synth CycleThrough(accepting)
```

Since we search for an accepting loop, inclusion and merging are unsound, but still complete. However, we can safely apply subsumption in NDFS. Without inclusion, the zone graph is infinite, so we are forced to resort to bounded synthesis, which only provides an under-approximation. Hence, we also use iterative deepening (BSID, Sec. 4.3). The depth limit is reached in 6 s.

[4] Inclusion and merging are sound and complete for reachability [7]. Inclusion applies maximal subsumption, while merging combines zones with exact convex hull.

```
imitator brp_RC.imi accepting.imiprop -depth-step=5 -depth-limit=25 -recompute-green
    4*TS + 3*TD >= TR & TS > 2*TD
OR TR + TD > SYNC + TS & TS > 2*TD
```

We could have searched even deeper for more liberal constraints, but it can be easily checked that this error constraint is equivalent to the complement of the safety constraint (within the initial condition), see Sec. 6.1, property **(R)**. Hence, we can conclude that we have already synthesised the exact constraint.

#### 6.3 Proper Liveness Properties

**GF(S_in).** Next, we will synthesise constraints for an actual liveness property, stating that the number of new sessions is infinite. We use Spot [15] to generate a Büchi automaton for the *negation* of this formula, and add the result as a monitor to the IMITATOR model, synchronising with the sender process. We add the constraints on correctness that we learned before to the initial constraints:

```
init := ... & SYNC >= TR & TS > 2*TD & TR > 4*TS + 3*TD
```

The following command tries to synthesize all parameters (within the initial constraint) for which an accepting loop is reachable, *i.e.* **GF** S_in is violated. We replaced subsumption by full inclusion, since otherwise IMITATOR gets lost in the infinite parametric zone graph. Recall that inclusion is complete but unsound for NDFS, so this provides an over-approximation of the constraints.

```
imitator -no-subsumption -comparison inclusion brp_GF_S_in_RC.imi accepting.imiprop
```

IMITATOR replies *False* in 1 second, so there is no reachable accepting cycle. Since this was an over-approximation, the result is conclusive: **GF** S_in holds under all parameter values inside this initial constraint. Note that, in principle, the property could be violated outside this initial condition. We can rerun the same experiment with the more general initial condition `TS > 2*TD`. IMITATOR confirms that the property still holds, but checking this larger space takes 19 s.

**G(S_in ⇒ F(S_ok ∨ S_nok ∨ S_dk)).** Using the same method, IMITATOR confirms in 16 s, that also this response property holds: every sessions start is followed by some indication.

```
imitator -no-subsumption -comparison inclusion brp_GSinFSdk.imi accepting.imiprop
```

**G(S_in ⇒ F(S_ok ∨ S_nok)).** Let us pretend that we forgot the indication *S_dk* (don't know). This time, we search for a symbolic counter-example (using the option `-witness`), under the initial condition `TS > 2*TD`.

```
property := #witness CycleThrough(accepting)
imitator brp_GSinFSnok.imi accepting_one.imiprop
```

As expected, IMITATOR finds a counter-example quickly (within 0.04s).

### 7 Conclusion

We presented and evaluated new semi-algorithms solving the liveness parameter synthesis problem for Parametric Timed Automata. We also introduced new soundness and completeness notions for such semi-algorithms. The new algorithms, based on BFS and Bounded Synthesis (BSID), at least enumerate all parameters leading to accepting lassos in the parametric zone graph. We showed that this property does not hold for all previous algorithms, which were based on NDFS. Our new algorithms are less sensitive to the particular search order than the previous NDFS algorithms, that could get stuck in some branch of the PZG.

Tab. 3 (left) shows the soundness and completeness status of all considered algorithms. Full inclusion and BS-$n$ can only provide an over-approximation (resp. under-approximation). The enumQ algorithm is complete, but never terminates (indicated by ××), so its partial soundness and completeness results are vacuous (indicated by (✓)). Although the problem is undecidable, one might still hope for an algorithm that enumerates all possible solutions (like enumQ, generating and testing all rational solutions) *and* produces a finite set of aggregate solutions (if it exists). The algorithm should terminate for practical cases.

Tab. 3 (right) shows the results of our algorithms for examples $\mathcal{A}_1$–$\mathcal{A}_6$. They either terminate with an exact (✓) or partial ((✓)) result, or diverge (×). In one case the addition of the layers strategy is needed to obtain a partial result ((L)).

Our last example shows another challenge to obtain a complete approach. The PZG of PTA $\mathcal{A}_6$ has a non-cyclic infinite path. It seems non-trivial to compute its limit constraint automatically. After $n$ steps, the parametric constraint is $p \geq n \times q$. So the limit constraint is $q = 0 \wedge p \geq q$.

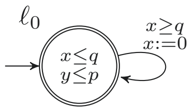

**Fig. 7.** PTA $\mathcal{A}_6$.

In order to handle cases where the set of solutions is not even a finite union of convex sets (Fig. 5), an entirely different representation of the solutions would be required.

Finally, exploiting the component-based structure of networks of PTA using a compositional approach, such as the one developed recently for fair paths in infinite systems [12], would be an exciting extension.

**Table 3.** Soundness and completeness properties of various algorithms.

| Algorithm | terminates | partially sound | partially complete | sound in the limit | complete in limit | complete for lassos | $\mathcal{A}_1$ | $\mathcal{A}_2$ | $\mathcal{A}_3$ | $\mathcal{A}_4$ | $\mathcal{A}_5$ | $\mathcal{A}_6$ |
|---|---|---|---|---|---|---|---|---|---|---|---|---|
| NDFS (enhanced) | × | ✓ | ✓ | ✓ | × | × | ✓ | × | × | (✓) | (L) | × |
| NDFS + inclusion | × | × | ✓ | × | × | × | ✓ | × | × | (✓) | (L) | × |
| BFS + SCC | × | ✓ | ✓ | ✓ | × | ✓ | ✓ | × | × | (✓) | (✓) | × |
| BSID | × | ✓ | ✓ | ✓ | × | ✓ | ✓ | ✓ | × | (✓) | ✓ | × |
| BS-$n$ (fixed bound) | ✓ | ✓ | × | ✓ | × | × | | | | | | |
| Naïve enumQ | ×× | (✓) | (✓) | ✓ | ✓ | ✓ | | | | | | |
